# Multi-well plate lid for single-step pooling of 96 samples for high-throughput barcode-based sequencing

**DOI:** 10.1007/s10544-024-00702-5

**Published:** 2024-02-28

**Authors:** Stéphanie Boder-Pasche, Mustafa Demir, Sarah Heub, Manon Garzuel, Réal Ischer, Daniel Migliozzi, Siegfried Graf, Noa Schmid, H. Baris Atakan, Daria Gudkova, Daniel Alpern, Riccardo Dainese, Bart Deplancke, Gilles Weder

**Affiliations:** 1 CSEM SA Centre Suisse d’Electronique et de Microtechnique, Jaquet-Droz 1, CH-2002 Neuchâtel, Switzerland; 2 Laboratory of Systems Biology and Genetics, Institute of Bio-Engineering & Global Health Institute, School of Life Sciences, EPFL, CH-1015 Lausanne, Switzerland; 3 Alithea Genomics, Biopôle, CH-1066 Epalinges, Switzerland; 4 Swiss Institute of Bioinformatics, CH-1015 Lausanne, Switzerland

**Keywords:** RNA sequencing, Barcoding, Multi-well plate, Sample pooling, Smart lid

## Abstract

High-throughput transcriptomics is of increasing fundamental biological and clinical interest. The generation of molecular data from large collections of samples, such as biobanks and drug libraries, is boosting the development of new biomarkers and treatments. Focusing on gene expression, the transcriptomic market exploits the benefits of next-generation sequencing (NGS), leveraging RNA sequencing (RNA-seq) as standard for measuring genome-wide gene expression in biological samples. The cumbersome sample preparation, including RNA extraction, conversion to cDNA and amplification, prevents high-throughput translation of RNA-seq technologies. Bulk RNA barcoding and sequencing (BRB-seq) addresses this limitation by enabling sample preparation in multi-well plate format. Sample multiplexing combined with early pooling into a single tube reduces reagents consumption and manual steps. Enabling simultaneous pooling of all samples from the multi-well plate into one tube, our technology relies on smart labware: a pooling lid comprising fluidic features and small pins to transport the liquid, adapted to standard 96-well plates. Operated with standard fluidic tubes and pump, the system enables over 90% recovery of liquid in a single step in less than a minute. Large scale manufacturing of the lid is demonstrated with the transition from a milled polycarbonate/steel prototype into an injection molded polystyrene lid. The pooling lid demonstrated its value in supporting high-throughput barcode-based sequencing by pooling 96 different DNA barcodes directly from a standard 96-well plate, followed by processing within the single sample pool. This new pooling technology shows great potential to address medium throughput needs in the BRB-seq workflow, thereby addressing the challenge of large-scale and cost-efficient sample preparation for RNA-seq.

**Graphical abstract:**

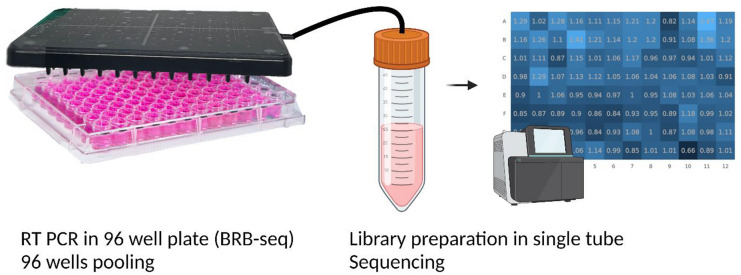

**Supplementary Information:**

The online version contains supplementary material available at 10.1007/s10544-024-00702-5.

## Introduction

Next-generation DNA sequencing (NGS) is the technology used for the determination of the order of nucleotides in targeted regions of DNA and entire genomes (from bacteria to humans) (Goodwin et al. 2016). NGS is a massively parallel sequencing technology that allows the generation of several millions of DNA reads in a cost-efficient and high-throughput manner. In particular, the cost of sequencing has drastically decreased in the last two decades, which led to, for instance, breaking the $1000/genome barrier and large-scale genomic projects involving the sequencing of several thousand genomes, such as the UK Biobank (Bycroft et al. 2018). Despite a growing interest to extend NGS to other applications such as RNA sequencing, DNA methylation or protein/DNA binding analysis, a major challenge relates to the need for laborious and expensive sample preparation processes, thereby limiting throughput and translational potential. RNA sequencing (RNA-seq) has become a standard for measuring genome-wide gene expression in biological samples (Stark et al. 2019). Combined with single-cell sequencing methods, RNA-seq enables the analysis of the transcriptome of individual cells, useful for studying cellular heterogeneity, cell fate determination, and tissue development (Jovic et al. 2022). Compared to legacy solutions such as microarrays, which are technically limited in the number of genes they can probe, RNA-seq provides a complete and unbiased gene expression readout. However, before the sequencing stage, RNA-seq involves several preparatory steps, where RNA must be extracted, converted to DNA, amplified, and quality-checked before it can finally be sequenced. As a result, the standard RNA-seq workflow requires several expensive reagents (enzymes and purification columns/beads) and significant manual effort. Consequently, despite its immense value for fundamental biological research, drug discovery, and biomarker applications, processing a large number of samples (i.e., more than 48) remains a significant challenge. Recently, several RNA-seq protocols have been published (e.g., PLATE-seq (Bush et al. 2017), DRUG-seq (Ye et al. 2018), and BRB-seq (Alpern et al. 2019)), which allow a substantial increase in the throughput and cost-efficiency of this NGS application. These technologies use early RNA barcoding, attaching sample-specific molecular tags to each sample during the reverse transcription. RNA barcoding allows any number of samples to be pooled right after reverse transcription and processed in one single tube for the rest of the workflow. This translates into a drastic reduction in reagent consumption and time used for experimental manipulation and decreases the risk of sample swapping during library preparation. Altogether, such protocols significantly empower RNA-seq technology with higher throughput and cost-efficiency, and the potential of RNA sample pooling has proven to optimize cost and statistical power in RNA sequencing experiments (Takele Assefa et al. 2020). However, a rate-limiting step for such protocols is a pooling step, where 96 or 384 samples need to be pooled in one single tube (Bush et al. 2017; Ye et al. 2018; Alpern et al. 2019). To date, the streamlining of this pooling process in terms of speed and automation has not yet been addressed beyond brute-force manual operation, which is slow and error-prone, or complex robotic procedures, which involve a relatively high cost and are not widely available.

Automated sample pooling from well plates has not yet been well addressed beyond solutions relying on liquid handling technologies or on customized pipetting robots (e.g. ASSIST PLUS technology from INTEGRA Biosciences 2021), for which the pooling throughput is limited either by the parallelization capacity of the instrument or its costs and footprint. Alternative methods to overcome this limitation rely on centrifugation (Sasagawa et al. 2018), or application of a negative pressure. For instance, the VBLOK200 Reservoir (ClickBio Inc.) is a commercially available disposable labware which consists of a V-shaped reagent reservoir suitable for collection of samples from a bottom-up oriented well-plate, during centrifugation. This appealing method enables quick and simultaneous collection of up to 1536 samples, but requires a dedicated centrifuge and has limited parallelization potential. Alternative platforms that are typically used to collect samples may also be adapted for a pooling operation. For instance, the devices for nucleic acid extraction (ADS Biotech, Inc) or simple vacuum manifolds (Qiagen, Inc) could be adjusted with ad hoc reservoirs for the pooled samples instead of individual wells of a multi-well plate. All these approaches require the transfer of the collected liquid into specific reservoirs for further storage or processing through, which may put constraints with regards to upscaling.

Innovative solutions have been developed to implement fluidic operations in multi-well plate formats, integrating microfluidic features in the well plates or on their lids, thereby enabling simple and automated sample preparation or sensing. Examples of lid devices integrate pneumatic actuation for perfusion (Huang et al. 2015) and perfusion combined with optical electrophysiology (Wei et al. 2020). Recently, commercial microfluidic lid platforms have been launched in the field of oxygen sensing (Lucid Scientific, Inc) and medium replacement for cell culture (Takasago Fluidic Systems). Furthermore, microfluidics integrated into well plates has also been developed and exploited for diverse applications. For example, gravity-based pumping was developed to automate perfusion for cell culture (Lee et al. 2007) with extended application for hanging drop-based culturing of spheroids (Kim et al. 2015). A similar approach was used to enable exposure of living tissues to drugs by creating a microfluidic well plate with an open-microfluidics window (Chang et al. 2024). More recently, an approach based on 3D printed microfluidics has provided standard well plates with microfluidic capabilities and well-well interconnections (Rauti et al. 2021). Such microfluidic approaches present many advantages in term of fluid control and liquid handling precision. However, their deployment at larger market scale is hindered by the technical complexity associated with the need for multi-material and multi-layer components integration, sometimes further requiring tight fluid sealing at the lid-plate interface.

**Fig. 1:**
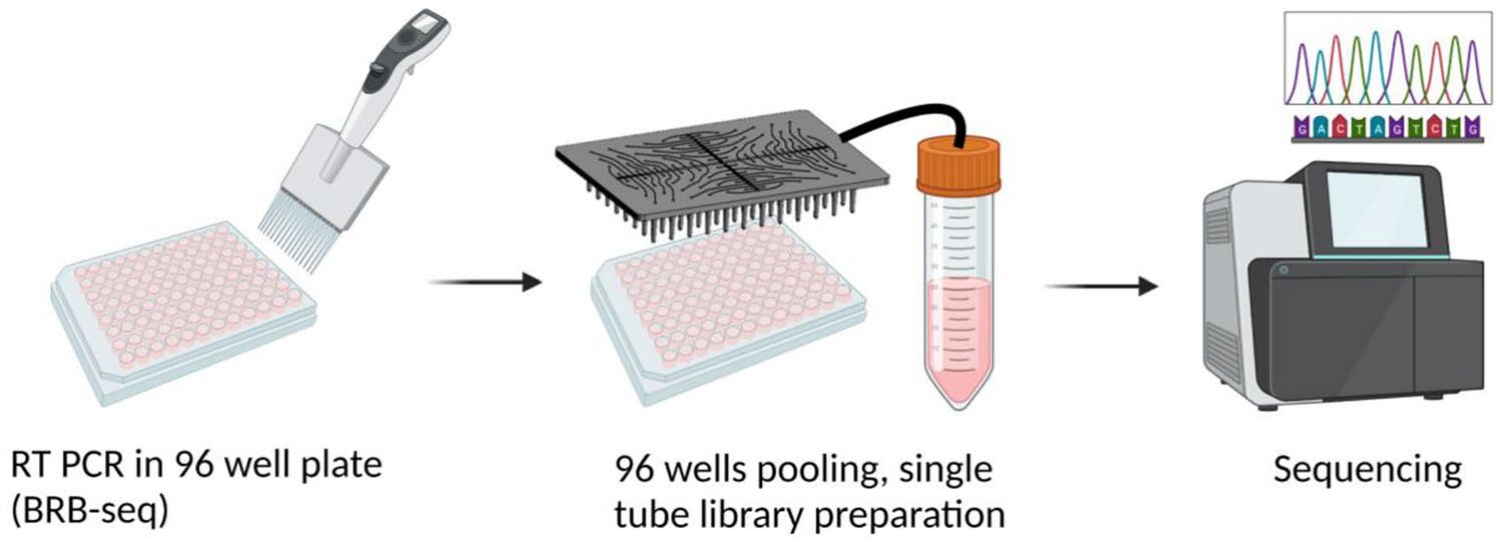
Schematics of the assay workflow: after filling the well-plate, the pooling lid is used to collect the content of all wells in a single step into a Falcon tube thanks to the negative pressure applied with a vacuum pump; the whole pool is subsequently processed for sequencing (created with BioRender.com)

This work proposes a simple benchtop solution for pooling the content of 96 well-plate wells in one automated step into a standard tube by means of a single-layer fluidic lid. Negative pressure applied to the lid’s unique outlet port enables to extract the content of a full plate in a single step within less than a minute. The design and fluid features of the pooling lids are first described, followed by performance in terms of liquid recovery. The potential for large-scale manufacturing process is demonstrated with injection molding, comparing the performances of a first milled prototype with a similar lid fabricated by injection molding. The approach is the applied to the pooling of cDNA barcodes and RNA-sequencing, demonstrating significantly decreased time and effort of sample pooling compared to manual operation. Finally, the upscaling potential of the approach is further highlighted with parallelization of four pooling lids, enabling the pooling of four plates in a single step. The proposed pooling method with the fluidic lid presents a small footprint not requiring large and costly equipment and opens the possibility for customization to increase throughput or combine with new functions, thereby making it a valuable tool for medium to large-scale barcode-based sequencing workflows and improving the cost per data-point. The assay components and the workflow are illustrated in Fig. 1.

## Materials and methods

### Fabrication of multiwell plate lid for pooling of 96-well plate

The pooling lid comprises microfluidic channels on the top and pins oriented towards the wells of the multi-well plate. The designs of the lids are described in the Section 3. A first prototype lid was fabricated in polycarbonate (PC) as a whole block and subsequently milled (P. Stadelmann SA) according to the selected fluidic design, with a channel width and depth of 200 µm. Metallic capillaries (Unimed, AISI 316L Tubing, 1 mm outer diameter, 0.2 mm inner diameter, 16 mm length) were press-fitted into circular through-holes (1 mm diameter, 3.5 mm deep) in the PC block. An additional metallic pin (1.27 internal diameter, length 10 mm) was glued at the side of the lid to enable connecting the lid channel input to the vacuum pump with soft fluidic tubing. Pressure-sensitive adhesive tape (ARflow® Adhesive Research) was used to close the microfluidic channels on top of the milled lids. Disposable pooling lids were injection molded in polystyrene (PS) (dna-Plasturgie Sàrl), after adapting the lid design according to molding process requirements. The fluidic channels on top of the injection molded lids were closed with a PS sheet (1.2 mm thick, Kahmann & Ellerbrock GmbH & Co.) sealed to the top lid surface by laser welding (Leister Technologies AG). A diode laser with a maximum laser power of 600 W and a wavelength of 940 nm was used. The laser beam had the form of a line with a length of 95 mm. The working distance from optic to clamping glass was 485 mm and the clamping force of 3′142 N was applied. The setpoint temperature was 90 °C and the lids were welded using 150 W laser power.

### Characterization of materials wetting properties

Surface wettability of the different materials composing the two lid prototypes was assessed. Static contact angle of di-water in air on the surface was measured with a drop shape analyzer (DSA 30 from Krüss). The method provides an average θ_CA_ from right and left angles formed by a 2 µL droplet deposited on the test surface. The average static water contact angle θ_M_ was calculated from five droplets for each surface sample.

### Fluidic and recovery tests

**Fig. 2:**
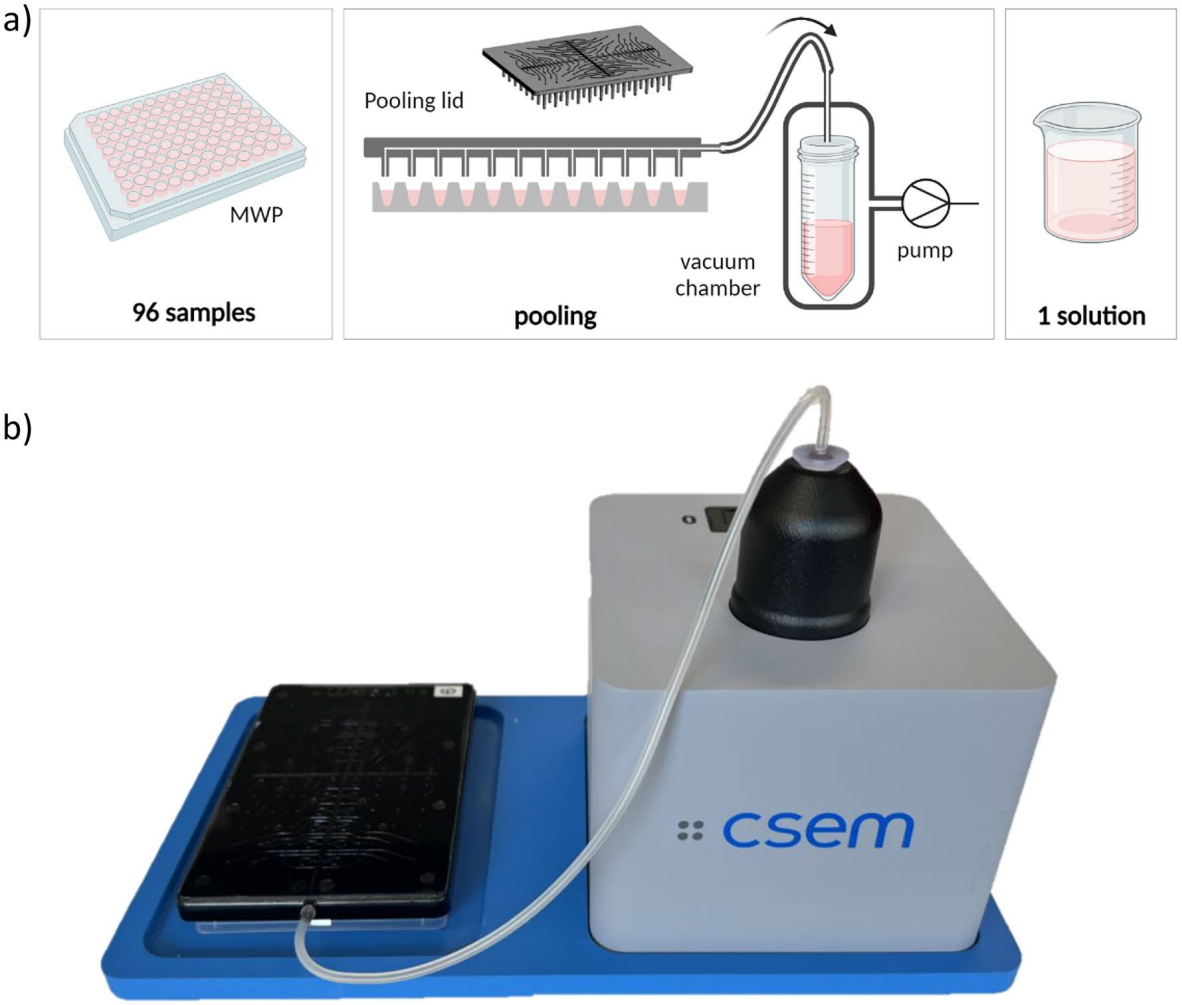
**a** Pooling process to transfer a multi-well plate into a single reservoir for batch processing (created with BioRender.com); **b** pooling setup comprising the multi-well plate, the pooling lid connected to a vacuum pump and to the reservoir

The pooling operation was performed as follows: the pooling lid was placed on a 96-well plate (conical bottom, ABgene) pre-filled with the sample. The lid was connected to the inlet port of the pooling reservoir using standard silicone tubing of 3 mm outer diameter and 1 mm internal diameter (Semadeni AG). The pooling reservoir consists of a 50 mL Falcon tube, which was placed in a 3D-printed custom-made chamber, closed with a sealing ring, and connected to the lid and to a vacuum pump. Negative pressure was applied to the closed system via an integrated diaphragm pump (Schwarzer Precision GmbH). The pump provides a maximum of -900 mbar vacuum with 6 L/min air flow. The pooling step was timed manually. Pooling efficiency was evaluated using a 96 well-plate. In order to ensure precise and reliable sample volume for the characterization experiments, the well-plates were initially filled with di-water using a Microlab STAR Liquid Handling System (Hamilton) equipped with eight pipetting heads and Venus Software Version 2. Liquid recovery was calculated from the amount of liquid collected in the tube, measured by weighing. The amount of liquid remaining in the well plate and in the lid was also measured by weighing. The pooling efficiency was evaluated for varying sample volumes (10 to 100 µL per well) and pooling time (30 s to 5 min) (Fig. 2).

### Cell culture and RNA extraction

Human HEK-293 cells were grown on 15 cm plates at 37 °C in Dulbecco’s modified Eagle’s medium (DMEM) supplemented with 10% fetal bovine serum and 1 × penicillin/streptomycin. Total RNA was isolated using Qiagen RNeasy Plus Mini Kit (Qiagen, ref 74134) according to the manufacturer’s instructions. Extra on-column DNase treatment was performed using RNA Clean& Concentrator kit (Zymo Research, ref R1014). The RNA concentration was determined using the NanoDrop One (Thermo Scientific), and its integrity was assessed using a Fragment Analyzer (Agilent).

### cDNA generation, pooling, and RNA-seq library preparation

The MERCURIUS BRB-seq library preparation kit (Alithea Genomics, Cat. 10813) was used to prepare barcoded cDNA from 100 ng of total RNA, 10 pmol of DNA barcodes (Alithea Genomics, 96 V5set0) in 20 µL of the final volume (per sample). Following a first-strand cDNA synthesis, samples were pooled either manually (with a 12-channel pipette, Integra, ref 3036) or through the custom microfluidic pooler (operated by a dedicated pressure regulator, OB1, ElveFlow). After pooling, cDNA was prepared according to the manufacturer’s instructions. For tagmentation, 20 ng of cDNA was used; the optimal number of amplification cycles was determined by qPCR. Libraries were purified and size selected using magnetic AmPure Beads (Beckman Coulter).

### RNA-seq library quality check and sequencing

Library concentration was measured by Qubit (ThermoFisher). The smear fragment size was determined by Fragment Analyzer (Agilent). Sequencing was performed on an Illumina NovaSeq 6000 with a High-output 100 cycle cartridge according to manufacturer instructions.

### Bioinformatic analysis

The sequencing reads from our own experiments were demultiplexed and aligned to the human hg38 genome using STARSolo. Correlation plots were generated using custom R scripts (version 3.3.1, https://cran.r-project.org/).

## Results and discussion

### Pooling lid design and fabrication

The pooling lid enables to collect and transfer the contents of the 96 wells of a multi-well plate into a single vial. The lid design relies on a network of 96 fluid channels joining in a larger collection channel that is guiding the sample from the wells to the lid outlet ports, tubing, then collection vial. The lid has vertical pins diving into each of the 96 wells of the plate to access sample at the bottom of the well. In the first few minutes of the pooling process, liquid is flowing through the sample channels in consistent flow conditions. Once air is introduced in the system, i.e. one well is empty, the flow in the corresponding channel is disrupted and an inhomogeneous air–liquid mixture is flowing through the complete channel network. Considering the dynamic air–liquid interface and unpredictable manufacturing-dependent features, the rationale behind the design relied on empirical considerations, such as ensuring sufficient pressure drop.

**Fig. 3:**
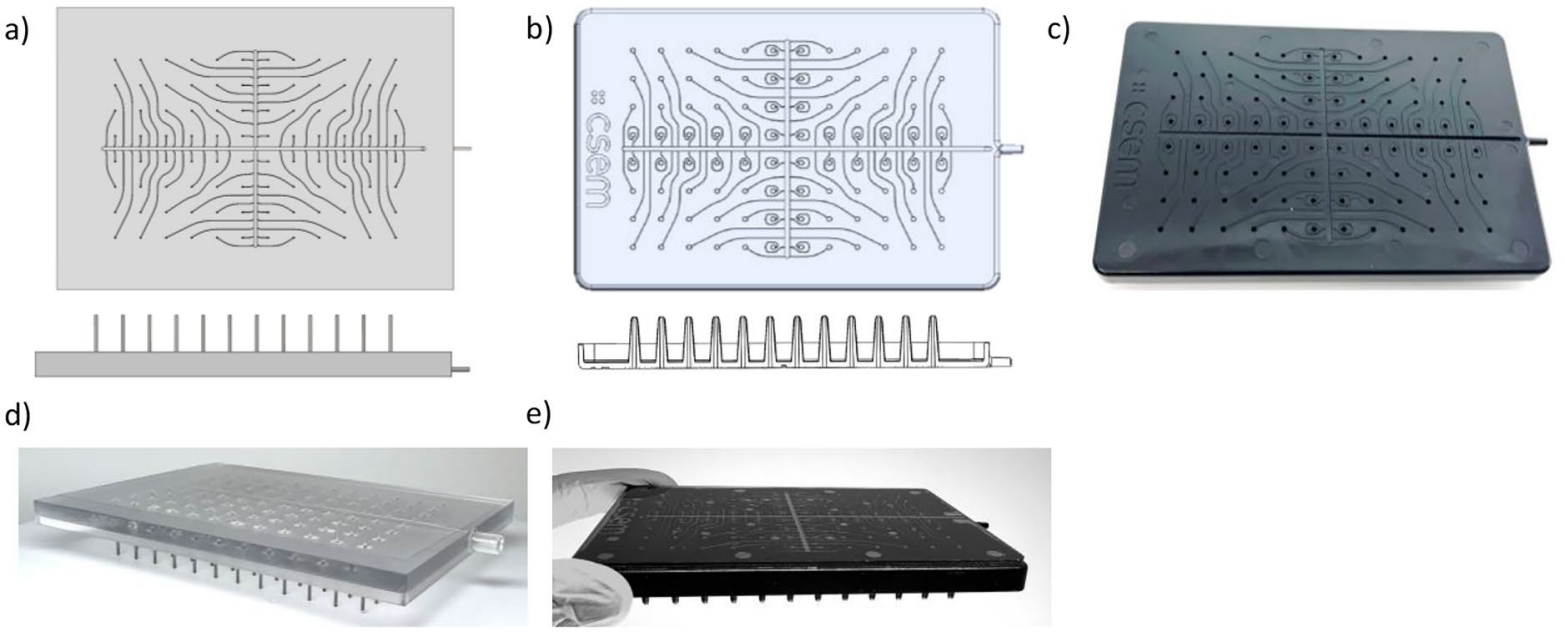
Microfluidic pooling lid: designs of (**a**) the PC milled prototype lid, **b** the PS injection molded lid, picture of (**d**) the PC milled prototype sealed with hydrophilic tape, and of the PS injection molded lid sealed with a PS plate (**e**) and (**c**) top view without sealing

On the milled prototype (Fig. 3a), the pins consist of 200 µm internal diameter and 16 mm long metallic capillaries. The fluidic channels present a 200 µm wide square section. They all join a 1.2 mm wide, 0.5 to 2 mm high central collection channel with negligible resistance. The design of the injection molded lid (Fig. 3b) was slightly modified according to processing constraints. The main limitation was related to the implementation of the pins. They consist of conical structures on the outside, facilitating unmolding of the part after the injection of plastic into the mold. The fluidic channel of the pin remains a cylinder, but the internal diameter could not be made smaller than 1.2 mm to ensure integrity of the high aspect ratio structures during unmolding procedure. The cross-section of the sample channels was also adjusted to facilitate unmolding, by forming a 1° angle between the channel wall and a vertical plan. The main differences between the milled and injection molded lids are the diameter of the pins, the length of the shortest channels, the material and surface properties of the lid.

### Pooling performances

The performances of the lids were tested with respect to pooling efficiency. The 96-well plates were filled with 20 µL water in each well. The lid was placed on the well plate, connected to the collection tube and to the vacuum pump. The volume per well and the pooling times were varied to assess their impact on pooling efficiency.

**Fig. 4:**
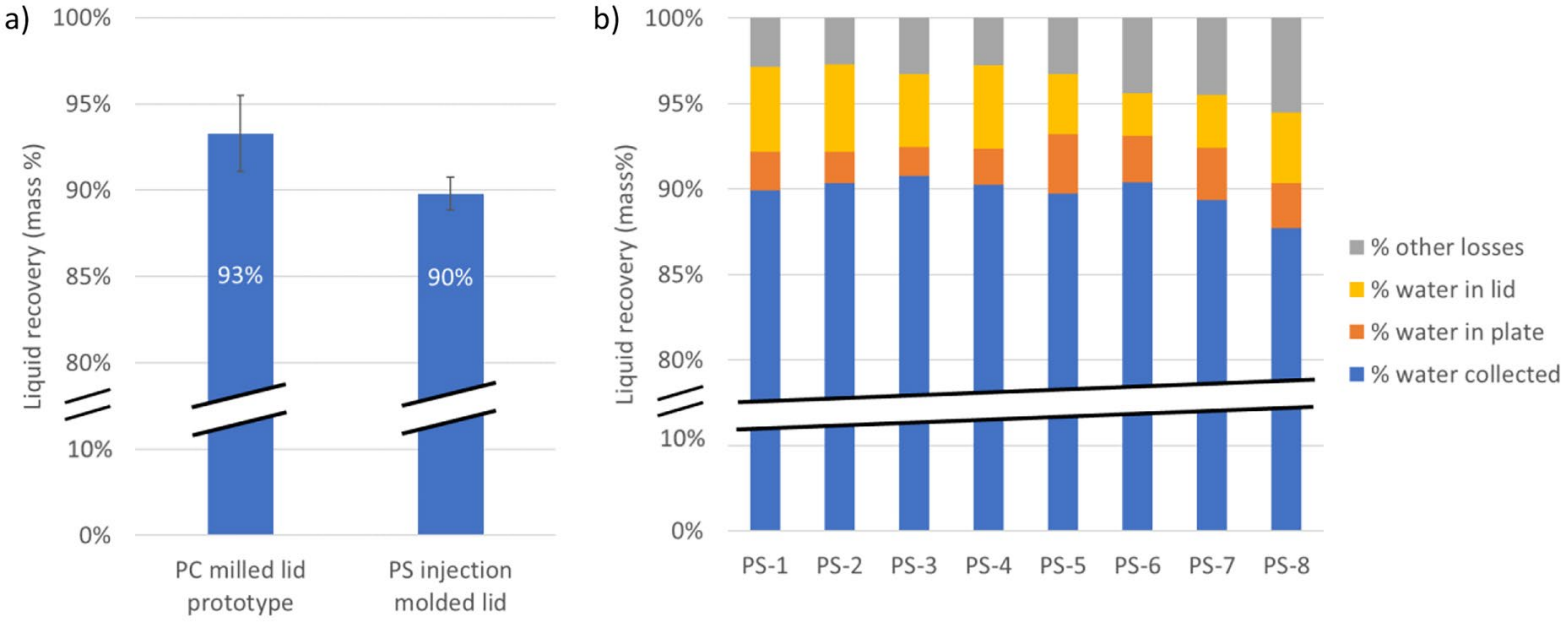
Pooling recovery data for the pooling of 20 µL/well in 2 min, **a** for the PC milled prototype lid (5 measurement replica with the same lid) and for the PS injection molded lids (average for 8 different lids, 1–2 measurement replica with each lid) (the error bars represent the standard deviation (*N* = 5 and 13, respectively)); **b** lid to lid reproducibility for the PS injection molded lid indicating the water recovery, water remaining in the lid, water remaining in the multi-well plate, and other losses (not measured), with < 1.5% pooling variation between two measurements with the same injection molded lid

Figure 4a compares the pooling performances of the PC milled lid prototype and of the PS injection molded lid. Results show comparable liquid recovery with both lids, despite design and materials variations. The larger pin diameter in the PS injection molded lid were expected to decrease the pooling performance, which is compensated by materials and fabrication variations. The liquid recovery was evaluated by comparing the weight of liquid in the plate before pooling and the weight of the liquid collected in the tube, and is expressed as the percentage of the final to the initial liquid weight. For the PC milled lid, the pooling performance leads to a recovery of 93% with 2% standard deviation, which drops to a recovery of 90% with 1% standard deviation for the PS injection molded lid. Deviations observed with the PC milled lid partly relate to incomplete drying of the lid between two measurements, as the same lid was used for all tests. On the contrary, eight different PS injection molded lids were used, with a maximum recovery difference of 3% between different lids (Fig. 4b). Repeated measurements with a single injection molded lid showed negligible variation. The lid-to-lid variation may either be related to small variation in the lid manufacturing, or to the pooling process itself.

As the pooling recovery is never reaching 100%, the losses were further investigated by weighting the remaining water in the lid and in the well-plate (Fig. 4b). Results show that sample losses are mostly related to liquid residues remaining in the lid (2 to 5%). Sample residues in the plate wells represent 2 to 3% of total sample volume. Additional losses (3 to 6%) would be attributed to liquid residues trapped in the tubing linking the lid to the collection vial, and potentially evaporation of a small volume of the sample contained in the well-plate.

**Fig. 5:**
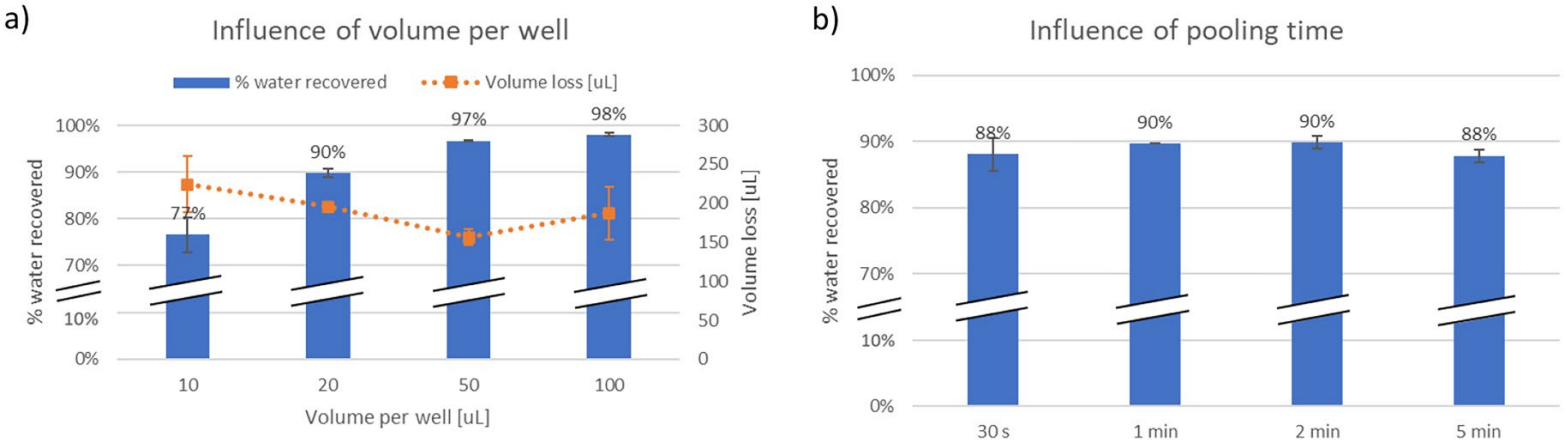
Influence (**a**) of the volume per well for 2 min pooling and (**b**) of the pooling time on the liquid recovery for 20 µL per well, measured with the PS injection molded lid (min 3 replicates per measurement) (the error bars represent the standard deviation (*N* = 3))

Then, the influence of sample volume was investigated by testing pooling performance of the injection molded lids for various starting volumes in the well-plate was evaluated with 10, 20, 50 and 100 µL (Fig. 5a). Results show an improved relative pooling performance for larger sample volumes. However, the absolute volume loss remains constant ranging from 150 to 230 µL per plate, or 1.5 to 2.4 µL per well in average. This experiment demonstrates that the sample losses are constant and independent from the initial sample volume.

The duration of the aspiration for the pooling step was also varied between 30 s to 5 min (Fig. 5b). Lower times were difficult to handle in comparison to the setup time, whereas for longer times liquid evaporation may interfere with the recovery data. Results show no significant influence of this parameter on the sample recovery. For following assays, the pooling step could be set to 30 s to minimize its duration.

**Fig. 6:**
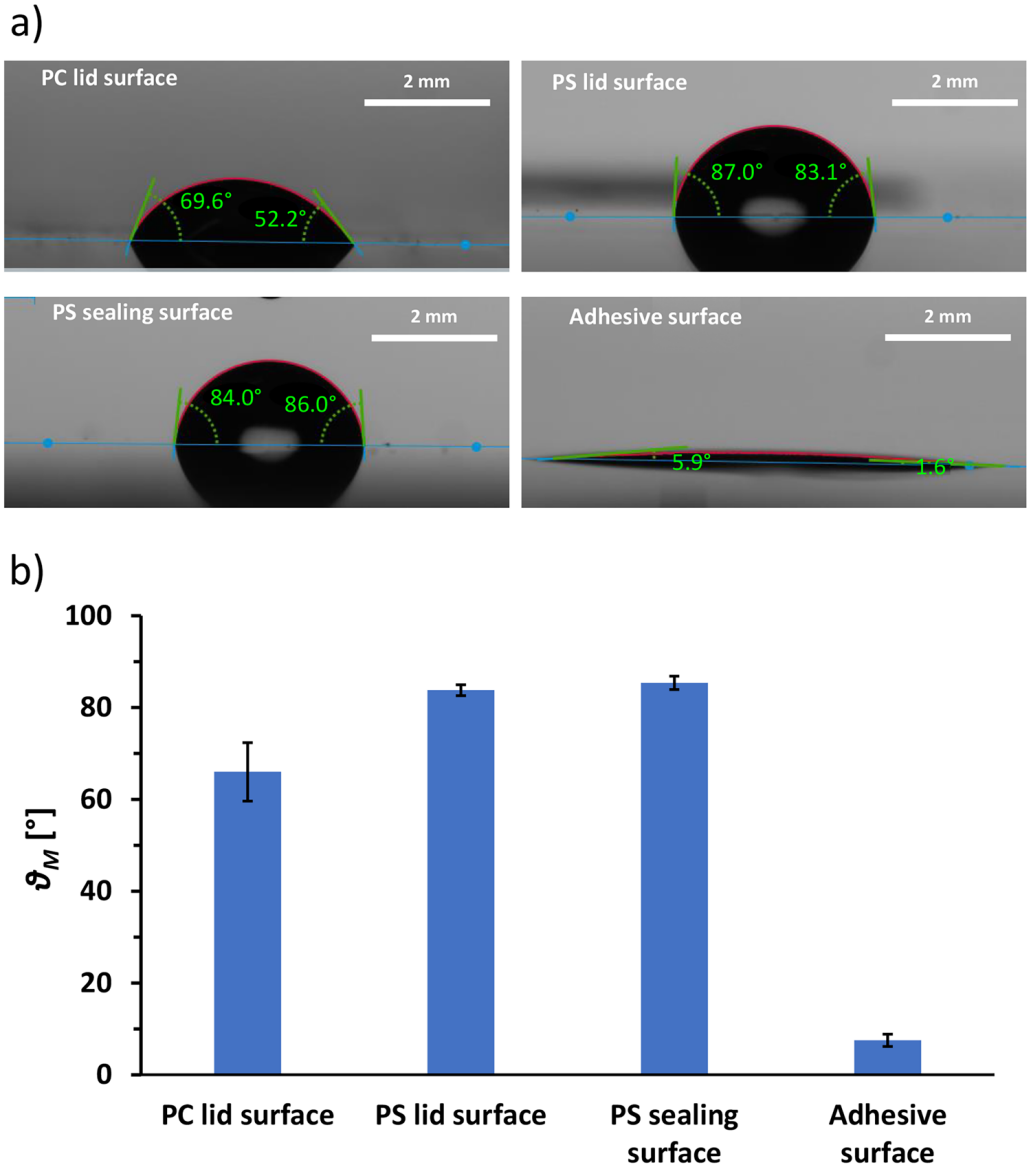
Water contact angle measured for the lid prototypes’ surfaces in contact with the sample: **a** representative pictures showing the 20 µL water droplet deposited on the test surface for the measurement showing right and left contact angles; **b** average water contact angle θ_M_ measured for each material (the error bars represent the standard deviation (*N* = 5))

With the proposed approach, liquid and air are mixed in varying proportions at the different locations of the lid and timepoints. The pooling efficiency is thus potentially more impacted by the channels’ surface properties and less by the channels’ geometries as it would be the case under classical liquid laminar flow conditions. The wetting properties of the channels of both lids were compared by measuring the water contact angle of the different surfaces in contact with the sample. For the milled lid, a pressure-sensitive hydrophilic adhesive tape is applied manually on the top of the PC channels. Thus, the hydrophilic surface of the film is in contact with the sample during the pooling operation. For the injection molded parts, a PS sheet is sealed around the channels by laser welding (Fig. 3e), leaving the content of the channels in contact only with PS surfaces. Surface wettability of the different materials in contact with the liquid was characterized by measuring the average static water contact angle θ_M_ (Fig. 6). The surface of the adhesive tape is highly hydrophilic as expected from product specifications. For the milled PC surface, θ_M_ is measured with a standard deviation of 10%. This wetting anisotropy is most likely linked to the surface roughness induced by the milling tool during fabrication. Both PS surfaces show similar wetting properties. Although the surface would still be considered hydrophilic, θ_M_ is significantly larger than the values obtained for the milled PC surface. Overall, the fluidic channels of the PS injection molded lid present a more hydrophobic surface compared to the assemble PC lid. This difference in wetting properties between both lids, associated with the different material selection for the fabrication, seems to compensate the larger pin diameter of the injection molded lid, which is expected to decrease the pooling efficiency.

The surface hydrophobicity might play a role in the sample losses encountered on the lid. As an inhomogeneous liquid–air mixture is flowing through the channels water residues would be trapped on the channel walls because of the increasing proportion of air in the mixture, flowing without encountering any resistance towards the vial and vacuum pump.

### Sequencing-based assessment of the pooling quality

A pooling of 96 cDNA samples from a corresponding 96-well plate was performed to assess the well-specific recovery. Pooling efficiency and accuracy were validated on a multiplexed RNA-seq experiment (BRB-seq) on 96 RNA samples (Alithea Genomics, Cat. 10813), by comparing the efficiency of manual pooling to pooling with the lid in 2 min. The efficiency of library amplification was defined as the main criterion for comparison (cDNA and library yield and a number of PCR amplification cycles) as well as correlation levels of obtained reads per barcode (every barcode should obtain 100/96 = 1.042% of all reads).

**Fig. 7:**
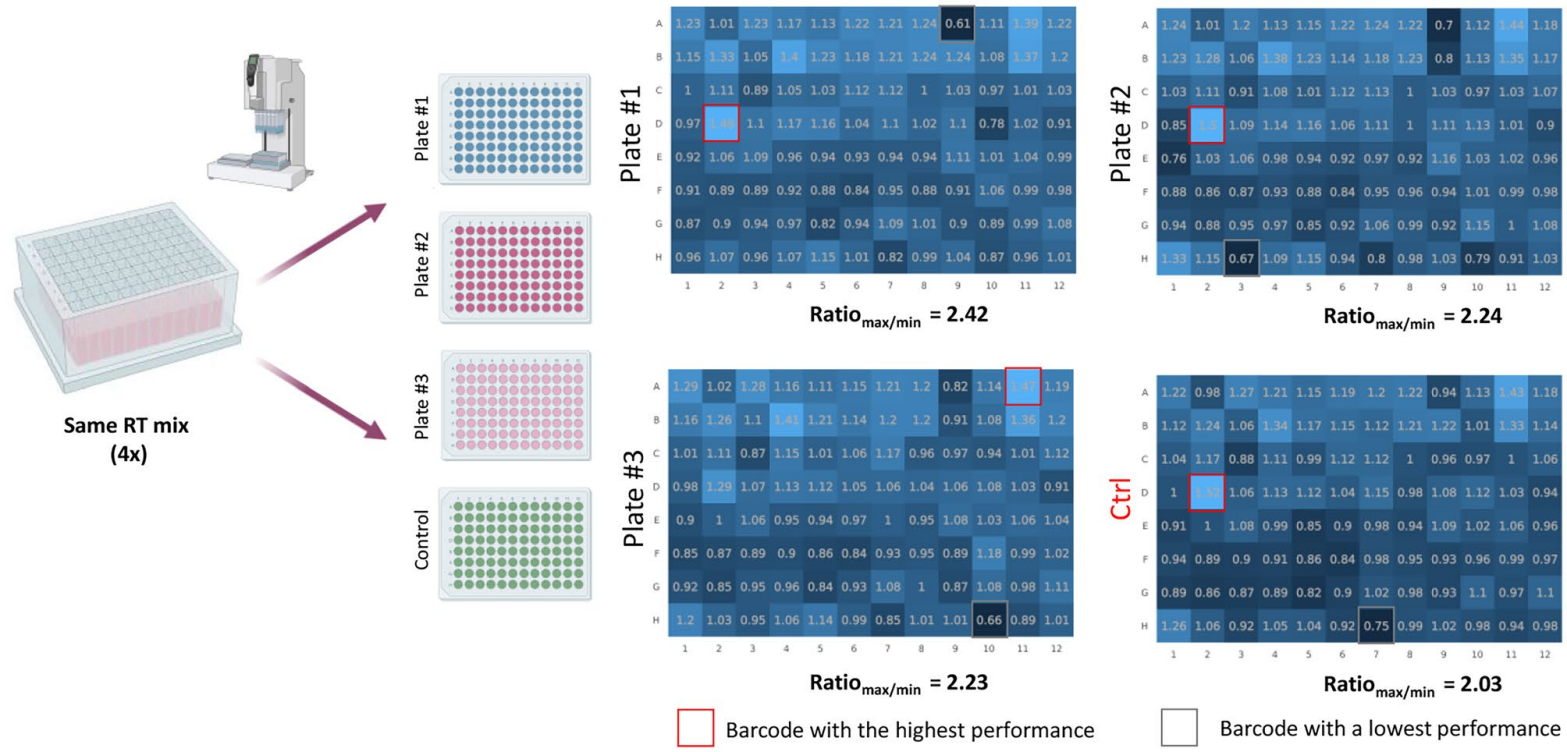
Sequencing data of the pooled samples comparing three 96-well plates pooled with PS injection molded pooling lids (PS-3, PS-4, PS-5) with a 96-well plate pooled manually (CTRL), showing for all tested lids an acceptable ratio (highest to the lowest) of obtained reads

For the well-to-well variation of the lids, RNA-sequencing of 4 libraries was performed. For this test, a sufficient amount of the master mix of barcodes and RNA was prepared in a single 96-well plate, followed by reverse transcription preparation. Then, using Vialfo 96 instrument, 20 µL (per well and per plate) was equally distributed in four 96-well plates for further pooling (Fig. 7). One plate (“Control”), was pooled manually, using a 12-channel mechanical pipette, while three others were pooled using three different injection molded lids (PS-3, PS-4, PS-5) and the automated pooling setup. The final pooled volume was in the expected range and did not vary significantly from plate to plate.

Libraries were prepared according to the manufacturer’s recommendations and did not show any variation either in the amplification cycle number or in the yield. Data analysis of the sequenced data has demonstrated a uniformity between obtained reads (Fig. 7). All obtained reads should be equally distributed between 96 barcodes, leading to 1.042% of reads per barcode. However, an up to threefold variation between highest and lowest barcodes is sometimes observed and deemed acceptable. As Fig. 7 shows, the tested lids, show a range of variation (max to min reads ratio) of 2.23–2.42 times, compared to 2.03 for the manual control. The measured reads show well-to-well standard deviations of 14%, 15% and 17% for the tested lids (PS-3, PS-4 and PS-5, respectively), compared to 13% for the manual control.

Overall, these results validate the presented automated pooling approach as a valid and rapid alternative to manual pooling, which can become especially valuable for medium to high-throughput experiments (e.g. high-throughput drug screening) involving large numbers of barcoded samples (hundreds to thousands of samples).

### Pooling lid technology for transcriptomics

Relying on a smart lid with 96 pins and fluidic channels, our pooling technology enables to simultaneously aspirate the liquid from all 96 wells of the multi-well plate. Combining the pooling lid with a vacuum pump, we demonstrated performances above 90% liquid recovery, with good reproducibility. The translation from a PC milled prototype to injection molded PS lids was achieved with some adaptation required by the manufacturing constraints and demonstrated upscaling potential without significant performance losses. The PS injection molded lid can therefore be used as disposable labware, thereby eliminating contamination risks by reusing a lid.

The pooling lid technology was tested for RNA-sequencing applications, highlighting the absence of significant biomolecule adsorption in the lid channels, and a homogeneous pooling from all wells with minimal losses. An overall recovery of at least 90% is sufficient for the tested RNA-sequencing assay. Combined with barcoding technology, the pooling lid demonstrates attractive potential for medium throughput genomics assays.

A compact and integrated setup enabled the pooling of one 96-well plate filled with 10 to 100 µL/well in 30 s in a single step. Manual pooling of the same multi-well plate with a pipet (single or multi-channel) would take a few minutes and lead to potential handling errors. Sample collection using a robotic liquid handling platform requires, in contrast, heavier infrastructure and, depending on the parameters, would not take less time unless parallelized. The small footprint and low cost of our standalone platform and simple components, not requiring more than the lid, the platform, and standard labware, makes is appealing for medium throughput applications and benchtop use.

**Fig. 8:**
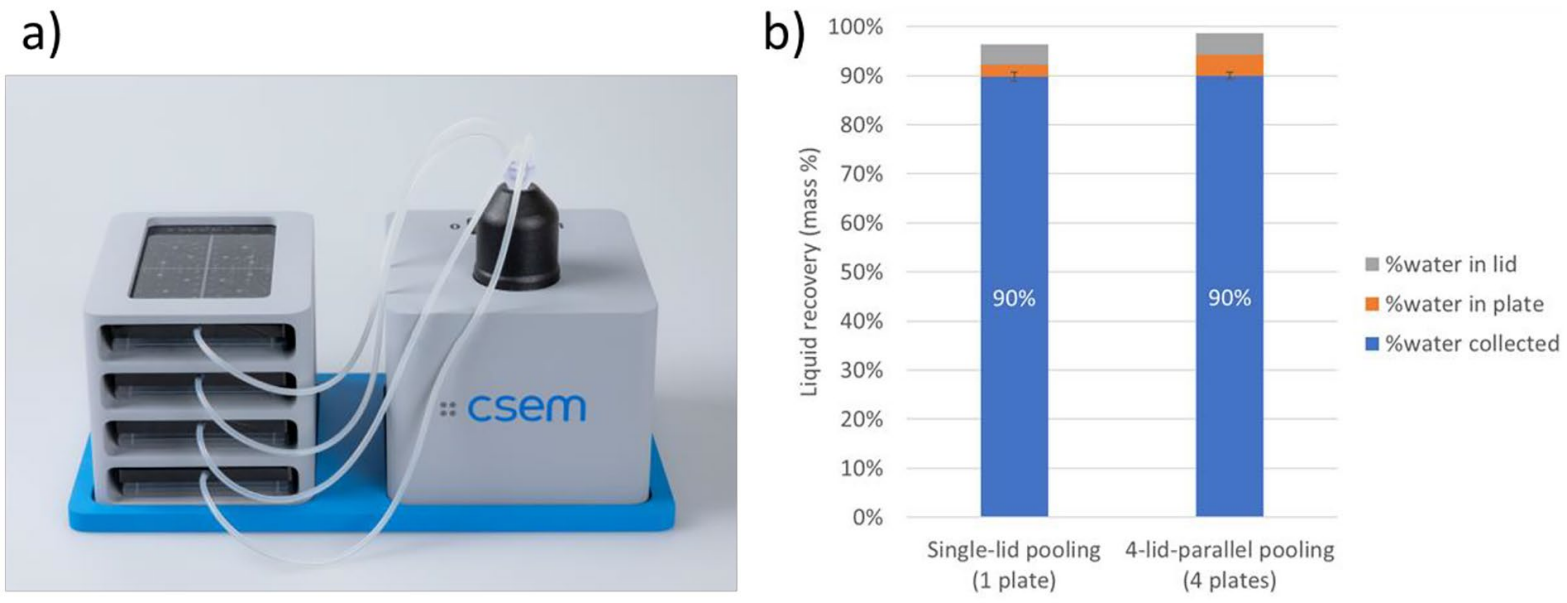
**a** Pooling setup for parallelization to process four plates at the same time; **b** pooling recovery in tube for single-plate pooling (Fig. 4a) compared to parallel pooling of four plates (the error bars represent the standard deviation (*N* = 13 and 4, respectively)), for a pooling time of 2 min. Shown are the liquid recovery and the remaining water in the plates and in the lids

The throughput of our pooling technology can be improved in two ways. On the first hand, the pooling lid design and fabrication can be adapted e.g., to a 384 well-plate format, and collect 384 samples in the single step with the same technology. Preliminary prototypes were designed and tested and demonstrated pooling efficiencies comparable to the lid for 96 wells for similar volume per well (SI, Fig. S1). Both the design and pooling process could be further optimized to enhance pooling efficiency, and the prototype fabrication could be scaled up with injection molding. This shows that the pooling concept can be adapted to different well plate formats, either by the number of wells or by the well/plate geometry. On the other hand, the setup can be adapted to pool several plates in parallel, by adding a multi-ways manifold on the tube. Preliminary data showed parallelization with up to four plates (Fig. 8). without impacting the pooling performance for 2 min pooling time.

The parallelization potential of the pooling lid approach was demonstrated by processing four plates in parallel with the same device (Fig. 8a). Four lids were placed onto four 96-well plates filled with 20 µL water in each well, and connected with the same standard silicone tubing (3 mm outer diameter and 1 mm internal diameter) to a fourfold custom 3D printed manifold placed on the collection tube. The pump was turned on for 2 min, and the liquid recovery was measured. Figure 8b shows the pooling efficiency for 2 min pooling of one plate compared to 2 min pooling of four plates in parallel. Similar pooling performances are achieved with one or with four lids, thereby decreasing the pooling time by a factor or four.

Additional functions could further be integrated with the pooling technology, such as e.g. concentration of the pooled sample with a PCR column placed in the Falcon tube, thereby reducing the number of transfer into different reservoirs and reducing sample losses.

## Conclusions

In this work, we have presented a single layer fluidic lid to perform fast and simultaneous pooling of 96 samples directly from a standard multi-well plate in a single step. Based on an injection molded smart pooling lid and a standalone platform, the technology is upscalable and easy-to-use, with a potential to integrate new functions. We evaluated the recovery of the samples and the potential for use in combination with BRB-seq technology for application to high-throughput sequencing. Our results indicate a pooling recovery of 90% with homogenous and unbiased collection of samples from the entire 96-well plate (2.23–2.45 max variations of reads) and sequencing-based benchmarking revealed comparable performance in terms of sample recovery and read density to manual pooling. Compared with alternative methods for pooling, our pooling lid can be interfaced and operated using common vacuum pumps accessible to any laboratory and connected to standard Falcon tubes, which avoid further sample transferring and increase the opportunity towards automation and high throughput. The small footprint, potential for parallelization and simplicity of the standalone benchtop technology make it appealing for medium throughput transcriptomics, and for genomics in general. Along with current developments of genomics kits relying on standard micro-well plate formats, further adaptations of the device can be envisioned, for example for incorporation into robotic liquid handling protocols for even greater throughout by parallelizing several lids/plates, which will open new perspectives for use of our tool in large screening facilities. Parallelization of the lids and redesign to other plate formats (e.g., 384-well plates) will increase the throughput efficiency. We believe that this approach based on a fluidic lid will be highly effective in providing both functionality and automation/throughput to barcode-based sequencing and other screening applications. We anticipate that this novel tool will enable large-scale transcriptomics and epigenomics and catalyze the widespread adoption of e.g., RNA-seq and ChIP-seq within the pharma/biotech sector, which, in turn, will unlock novel applications toward much larger untapped markets such as the ones of drug and biomarker discovery.

### Supplementary Information

Below is the link to the electronic supplementary material.

Supplementary file1 (PDF 384 KB)

## Data Availability

The data that supports the findings of this study are available from the corresponding author on request.
