# Supplementary material for: Multi-well plate lid for single-step pooling of 96 samples for high-throughput barcode-based sequencing
Source: Biomed Microdevices. 2024 Feb 28;26(2):18. doi: 10.1007/s10544-024-00702-5 (PMC10902082; doi:10.1007/s10544-024-00702-5)
Supplement: Supplementary file 1 — Supplementary file1 (PDF 384 KB) [file 10544_2024_702_MOESM1_ESM.pdf]

Supplementary Information (SI) to *Biomedical Microdevices*

Multi-well plate lid for single-step pooling of 96 samples for high-throughput barcode-based sequencing

Stéphanie Boder-Pasche[*1], Mustafa Demir[2,4], Sarah Heub[1], Manon Garzuel[1], Réal Ischer[1], Daniel Migliozzi[1], Siegfried Graf[1], Noa Schmid[1], H. Baris Atakan[1], Daria Gudkova[2,4], Daniel Alpern[2,3,4], Riccardo Dainese[2,3,4], Bart Deplancke[2,3] and Gilles Weder[1]

[1] CSEM Centre Suisse d'Electronique et de Microtechnique, Switzerland
[2] Laboratory of Systems Biology and Genetics, Institute of Bio-engineering & Global Health Institute, School of Life Sciences, EPFL, CH-1015 Lausanne, Switzerland
[3] Swiss Institute of Bioinformatics, CH-1015 Lausanne, Switzerland
[4] Alithea Genomics, Biopôle, 1066 Epalinges, Switzerland

[*] *Corresponding author: Stéphanie Boder-Pasche, CSEM SA, Jaquet-Droz 1, CH-2002 Neuchâtel, Switzerland, email: stephanie.boder@csem.ch, ORCID: 000-0003-0934-2026*

This document provides additional information complementing the data shown in the main manuscript, which relies on preliminary data on prototypes in development.

SI1. Upscaling to 384 well plate format

The pooling lid approach can be extended to different plate formats, such as e.g. 384-well plates, provided a specific adapted lid design. Aiming at increasing the throughput, the pooling of 384-well V-shaped PCR plates with the pooling lid approach was demonstrated with the design and fabrication of two new lid prototypes with a dedicated lid design (Fig. S1a) (channel width 200 µm). Preliminary prototypes were fabricated by milling (Fig. S1 b) using the same fabrication approach than for the 96-well lid, with metallic capillaries (Unimed, AISI 316L Tubing, 1 mm outer diameter, 0.2 mm inner diameter) press-fitted into circular through in the PC block. Aiming at a design compatible with injection molding, a 3D printing prototype (Protolabs, Ltd.) was fabricated for proof-of-concept (Fig. S1 c). Pressure-sensitive adhesive tape (ARflow® Adhesive Research) was used to close the microfluidic channels on top of both lids.

The lids were tested with a 384-well plate (conical bottom, ABgene) filled with 10 µL water per well, showing 72 and 67% liquid recovery with the milled and the 3D printed lid, respectively (Fig. S1d). The milled lid for 384 well plates therefore showed comparable pooling performance as the 96W lid for pooling of 10uL/well (72%, see Fig. 5 of the Manuscript). These prototypes are in development and have not yet been optimized for better pooling efficiency. They however demonstrated compatibility of the pooling approach with the 384-well plate format. Priority at that stage was not yet to manufacture this lid with injection molding, although this was considered in the design. This is now being considered in future work. The proof-of-concept with the injection molded lid for 96-well plate indeed paves the way for future upscaling, such as e.g. with 384 well-plates.

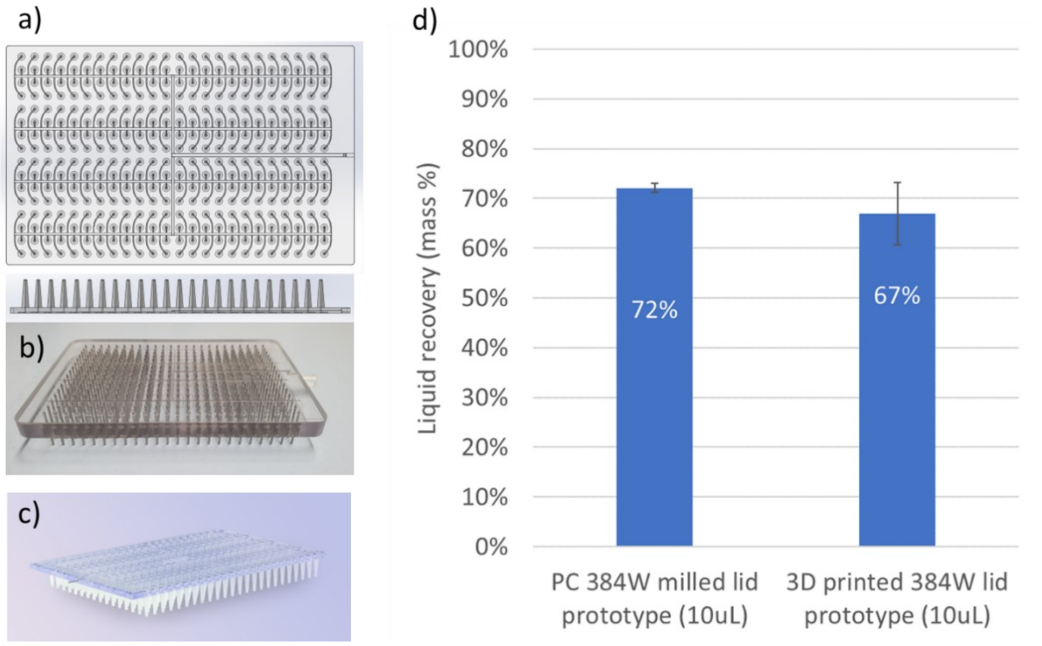

Fig. S1 Lid design (a), milled (b) and 3D printed (c) lid prototypes for the pooling from V-shaped 384 well plates; (d) pooling recovery data for the pooling of 10 µL/well in 2 min for the PC milled and for the 3D printed prototype lid and for the 3D printed prototype lids (the error bars represent the standard deviation (N=3)).

SI2. Comparison of pooling lid approach with alternative techniques

The main differences between pooling techniques are related to their cost, use, automation potential and parallelization, and are summarized in Table S1.

Table S1. Qualitative comparison between techniques for the pooling of multi-well plates

Technique	Equipment cost	Cost per data point	Footprint	Automation potential	Process time	Parallelization (multiple plates)
**Manual (single or multi-pipet)**	Low	High	Small	Low	High	Low
**Pipetting robot**	High	Medium	Large	High	Low	High
**Centrifuge**	Medium	Low	Medium	Low	Medium	Medium
**Pooling lid**	Low	Medium	Small	Medium	Low	High
